# Prevalence and determinants of erectile dysfunction in Santos, southeastern Brazil

**DOI:** 10.1590/S1516-31802002000200005

**Published:** 2002-03-02

**Authors:** Edson Duarte Moreira, Walter Jorge Bestane, Elaine Bestane Bartolo, João Antônio Saraiva Fittipaldi

**Keywords:** Erectile dysfunction, Impotence, Prevalence, Risk factors, Brazil, Disfunção erétil, Impotência, Prevalência, Fatores de risco, Brasil

## Abstract

**CONTEXT:**

Recent population-based surveys suggest that the prevalence of erectile dysfunction is between 30% and 56% among men over the age of 40. Most of these studies, however, are from the United States or Europe. We need estimates of erectile dysfunction from samples of Brazilian populations, as societies that differ ethnically, culturally, and economically may also differ with respect to potential risk factors for erectile dysfunction.

**OBJECTIVE:**

To determine the prevalence of erectile dysfunction and its potential correlates.

**SETTING:**

Santos, State of São Paulo.

**DESIGN:**

Cross-sectional study.

**PARTICIPANTS:**

A population-based sample of men aged 40-70 years. Out of 718 men invited, 342 (47.6%) returned a completed questionnaire.

**MAIN MEASUREMENTS:**

Data on demographic variables, medical history, lifestyle habits and degree of erectile dysfunction.

**RESULTS:**

The prevalence of any degree of erectile dysfunction was 45.9% (minimal, 33.9%; moderate, 8.5%; complete, 3.5%) and increased with age. In bivariate age-adjusted analyses comparing men with no erectile dysfunction or minimal erectile dysfunction with those with moderate or complete erectile dysfunction, histories of diabetes or hypertension, depressive symptoms, heavy smoking and obesity were significantly associated with increased prevalence of erectile dysfunction, whereas moderate alcohol consumption was inversely associated with erectile dysfunction. In the multivariate model, age was a strong predictor of erectile dysfunction, while history of diabetes or hypertension and heavy smoking remained significantly associated with increased prevalence of erectile dysfunction.

**CONCLUSION:**

We found higher prevalence of erectile dysfunction (45.9%) among men older than 40 years old in Brazil. The variables associated with erectile dysfunction may alert physicians to patients who are at risk of erectile dysfunction as well as offer clues to the etiology of erectile dysfunction. Physicians should routinely ask their patients about sexual health and erectile dysfunction.

## RESUMO

**CONTEXTO:**

Inquéritos populacionais recentes sugerem que a prevalência de disfunção erétil é de 30% a 56% entre homens acima de 40 anos. Entretanto, a maioria desses estudos foi realizada nos Estados Unidos ou Europa. É preciso estimar disfunção erétil a partir de amostras da população brasileira, uma vez que sociedades com diferenças étnicas, culturais e econômicas também podem diferir com respeito a fatores de risco para disfunção erétil.

**OBJETIVO:**

Este estudo visa determinar a prevalência de disfunção erétil e potenciais fatores correlatos em Santos, sudeste do Brasil.

**TIPO DE ESTUDO:**

Estudo de prevalência ou transversal.

**LOCAL:**

Cidade de Santos, Estado de São Paulo.

**PARTICIPANTES:**

Um inquérito, com questionário auto-administrado, enviado pelo correio, foi realizado numa amostra de base populacional. Monitores treinados distribuíram o questionário da pesquisa a homens de 40 a 70 anos, num envelope pré-endereçado e selado. Dos 718 homens convidados, 342 (47,6%) enviaram de volta o questionário completo.

**VARIÁVEIS ESTUDADAS:**

Os dados foram coletados por meio de questionário estruturado com informações referentes a características demográficas, história médica pregressa e hábitos de vida. O grau de disfunção erétil foi categorizado como "nenhum", "mínimo", "moderado" ou "completo" de acordo com a capacidade do indivíduo em "ter e/ou manter uma ereção satisfatória para relações sexuais". Análises bivariada ajustada para idade e multivariada foram realizadas para calcular a razão de chances (*odds ratio*) e intervalos de confiança de 95%.

**RESULTADOS:**

A prevalência de algum grau de disfunção erétil foi de 45,9% (mínima, 33,9%; moderada, 8,5%; completa, 3,5%), aumentando com a idade. Na análise bivariada, comparando-se os homens sem ou com disfunção erétil mínima versus aqueles com disfunção erétil moderada ou completa, história de diabetes ou hipertensão, sintomas de depressão, tabagismo (^3^ 40 cigarros/dia) e obesidade (índice de massa corpórea > 30,0 kg/m^2^) foram significativamente associados com prevalência aumentada de disfunção erétil (p < 0,05), enquanto que o consumo moderado de álcool foi inversamente associado com disfunção erétil. No modelo multivariado, idade foi um preditor forte de disfunção erétil, enquanto história de diabete ou hipertensão e tabagismo permaneceram significativamente associados com disfunção erétil.

**CONCLUSÃO:**

Consistentemente com outros estudos de prevalência de disfunção erétil, encontramos alta proporção (45,9%) de homens acima de 40 anos no Brasil com algum grau de disfunção erétil, aumentando a prevalência e a gravidade de disfunção erétil com a idade. As variáveis de estilo de vida e de condições de saúde associadas à disfunção erétil podem alertar os médicos acerca de pacientes sob risco de disfunção erétil, como também oferecem pistas quanto à etiologia da disfunção erétil. Profissionais de saúde deveriam sempre perguntar aos seus pacientes sobre saúde sexual e disfunção erétil, uma vez que uma grande proporção da população experimenta tais problemas.

## INTRODUCTION

Erectile dysfunction, defined in 1992 by a National Institutes of Health Consensus Panel^1^ as the persistent inability to attain or maintain an erection sufficient for satisfactory sexual function, has recently been the focus of public attention due to the development of a novel effective oral therapy.^2^ The term "impotence" with its derogatory and non-specific meaning was then substituted by the more precise term of erectile dysfunction. This sexual disorder is a common problem affecting the well-being of aging men, as recent population based studies indicate that 30 to 56% of men aged 40 to 70 years old have some degree of erectile dysfunction.^3-9^ Within this age range, it has been estimated that 18 million men in the United States^10^ and 9 million men in Brazil are affected by some degree of erectile dysfunction.^11^ Although not life-threatening, erectile dysfunction should not be regarded as a benign disorder, as it can have a strong negative effect on interpersonal relationships, well-being and quality of life.^12^

Epidemiological data on erectile dysfunction used to be relatively scant, but recent pharmacological advances that offer significant therapeutic potential for the treatment of male erectile disorder^2,13,14^ have sparked professional and public interest in this sexual dysfunction, and have generated a wealth of data on this topic.^3-9^ Population-based data concerning the prevalence, determinants, and consequences of erectile dysfunction from different countries may be very useful, as societies that differ ethnically, culturally, and economically may also differ with respect to potential risk factors for erectile dysfunction. The present study has attempted to determine the prevalence of erectile dysfunction and its potential demographic, medical and lifestyle correlates in southeast Brazil by using data from a population-based survey. It has also addressed the role of age-related health indexes and sociocultural predictors as determinants of erectile dysfunction.

## METHODS

### Study Sample

The study sample consisted of respondents to a cross-sectional mail survey of health status and lifestyle variables among men aged 40 to 70 years old, conducted between October 1999 and March 2000. In an attempt to increase the response rate, we used an in-person delivery strategy to distribute the questionnaires. This approach will be the focus of a separate manuscript. In summary, questionnaires were distributed by College students (study pro-moters) to men aged 40 to 70 years. First, they briefly explained to the potential participant how important the study was, emphasizing the anonymous nature of the survey and its brevity. Then, each subject who agreed to participate received a sealed package containing a questionnaire in a preaddressed stamped envelope and a letter stating the purposes of the study and thanking him for participating. The men were instructed to complete the questionnaire alone and then drop the envelope in a mailbox. There were 131 study promoters, with each one distributing five questionnaires on average. Out of 718 subjects invited, 659 (91.2%) agreed to participate and 342 of them eventually mailed the questionnaire back (a response rate of 47.6%).

### Data Collection

Data were collected via a self-administered standardized questionnaire that included items on demographics, modifiable lifestyle characteristics, general health (self-reporting of medical conditions), sexual behavior, satisfaction with sex life, frequency of intercourse and penile erections. Presence of depression symptoms was assessed by the shortened (five-item) Center for Epidemiological Studies Depression Scale (CES-D), with scores ranging from 5 to 20, higher scores denoting more depressive symptoms.^15^ With regard to erectile dysfunction assessment, the subjects were requested to choose one category that best described them, such as always, usually, sometimes or never being able to achieve and maintain an erection satisfactory for sexual performance. The answers were then used to classify the respondents into one of the following categories: none, minimal, moderate or complete erectile dysfunction, respectively. Thus, erectile dysfunction presence was assessed by the individual himself in terms of a single global question derived directly from the erectile dysfunction definition proposed at the National Institutes of Health Consensus conference.^1^ The statistical validation of this subjective approach was established in the Massachusetts Male Aging Study calibration study.^16^ In addition, Derby et al.^17^ validated the use of a single self-assessment question against two well-established erectile dysfunction measures, the International Index of Erectile Function^18^ and the Brief Male Sexual Function Inventory.^19^

### Statistical Analysis

In all bivariate and multivariate analyses, erectile dysfunction was categorized as "none" or "minimal" versus "moderate" or "complete". For each independent variable, crude and age-adjusted bivariate odds ratios (OR) and 95% confidence intervals (CI) were calculated. Statistical significance (two-tailed p-value ≤ 0.05) was assessed by *x^2^* for categorical variables and by Student's t-test and ANOVA for continuous variables. In the multivariate logistic regression, full models were made to fit, and then non-significant (p > 0.1) variables were eliminated in a stepwise backward elimination algorithm, with the least significant being first, in order to determine the final model. Exceptions were made for the medical variables, which were forced into the model as being of primary interest in the study.

## RESULTS

Selected demographic, medical, and lifestyle characteristics of the study population are presented in Table 1. The mean age (standard deviation) of men in our sample was 49.1 (8.4) years. The participants in our study had higher educational attainment than the average for Brazilian men.^20^ The frequency distribution of age, religion and marital status in our sample was comparable to that found in Brazil.^20^ The most common medical conditions reported included hypertension in 16.7%, gastrointestinal ulcer in 13.7% and diabetes in 7.6% (Table 1). Most responders were sexually active, with 315 (92.1%) men reporting sexual intercourse in the past six months. Table 2 shows that self-rated erectile dysfunction was characterized in our data as lower monthly rates of sexual activity and erection, higher frequency of erectile difficulty, and lower satisfaction with sex life and partner.

**Table 1:** Baseline characteristics of 342 men, Santos, Brazil, 1999-2000

Characteristics	
**Physical measurements (Mean** ± **standard deviation)**	
Age, years	49.1 ± (8.4)
Height (m)	1.74 ± (0.07)
Weight (kg)	81.1 ± (15.0)
Body Mass Index (kg/m^2^)	26.8 ± (4.34)
**Marital status, n = 342 (%)**	
Married or living with partner	274 (80.1)
Divorced	39 (11.4)
Single	21 (6.1)
Widowed	8 (2.3)
**Education, n = 338 (%)**	
Primary school completed or less	101 (29.9)
High school (incomplete or graduate)	68 (20.1)
College (incomplete or graduate)	169 (50.0)
**Religious affiliation (%)**	
Catholic	227 (66.4)
Spiritualist	41 (12.0)
Protestant	37 (10.8)
Other	15 (4.4)
None	22 (6.4)
**Smoking habit (%)**	
Never smoked	121 (35.4)
Past smokers	136 (39.8)
Current smokers	85 (24.9)
**Alcohol consumption, n = 337 (%)**	
None	118 (35.0)
≤ 3 drinks/day	170 (50.5)
> 3 drinks/day	49 (14.5)
**Medical Conditions (%)**	
Hypertension	57 (16.7)
Peptic ulcer	47 (13.7)
Diabetes	26 (7.6)
Benign prostate hyperplasia	25 (7.3)
Depression	21 (6.1)
Heart disease	16 (4.7)

**Table 2:** Sexual activity and satisfaction according to category of erectile dysfunction in 342 men, Santos, Brazil, 1999-2000

	Erectile Dysfunction			
	None (n = 185)	Minimal (n = 116)	Moderate (n = 29)	Complete (n = 12)
No sexual activity within last 6 months (%)	2	3	14	67
Sexual activity (median frequency/month)*	12	8	8	3
Full erection (median frequency/month)	16	12	8	2
Awaken with erection (median frequency/month)	10	10	4	1
Satisfaction with sex life †	4.1	3.8	3.2	2.1
Satisfaction with partner †	4.2	4.0	3.8	2.5
Partner satisfaction †	4.3	4.1	3.7	1.8

* Among those reporting some sexual activity within the last 6 months

† Mean on scale from 1 (extremely dissatisfied) through 5 (extremely satisfied).

### Prevalence and age dependence

**Figure 1:**
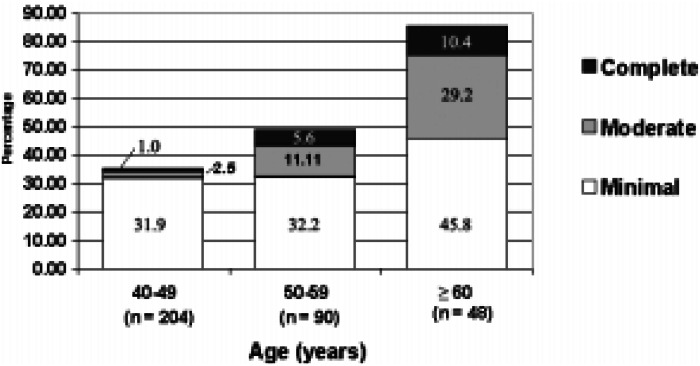
Association of subject age with degree of erectile dysfunction in 342 men, Santos, Brazil, 2000.

Overall, 45.9% (95%, CI = 40.5-51.4) of the men reported some degree of erectile dysfunction; the frequencies of minimal, moderate and complete dysfunction were 33.9% (95%, CI = 28.9-39.2), 8.5% (95%, CI = 5.812.0) and 3.5% (95%, CI = 1.8-6.0), respectively (Table 3). The relationship of erectile dysfunction prevalence to the age of study subjects is depicted in Figure 1. The combined prevalence of moderate and complete erectile dysfunction increased with age, from 3.4% in men aged 40-49 to 16.7% in men aged 50-59 to 39.6% in men aged 60-70. An estimated 65% of the men had no erectile dysfunction at age 40 years, decreasing to 15% at age 60 years. The association between age and erectile dysfunction was always statistically significant (p < 0.0001) when tested by multivariate analysis along with other possible predictors of erectile dysfunction.

**Table 3:** Prevalence of erectile dysfunction among 342 men in Santos and in another population-based survey in Brazil

Degree of erectile dysfunction	Prevalence (%)	
	Study sample (n = 342)	BSSB (n = 1,170) ‡
Absent	54.1	53.8
Present	45.9	46.2
Minimal	33.9	31.5
Moderate	8.5	12.1
Complete	3.5	2.6

‡ Brazilian Study of Sexual Behavior (BSSB)^11^

### Bivariate age-adjusted associations

The age-adjusted bivariate associations between erectile dysfunction and potential covariates are shown in Table 4. None of the sociodemographic variables was significantly associated with moderate or severe erectile dysfunction. Obesity, defined as a body mass index greater than 30.0 kg/m^2^, was significantly associated with increased prevalence of erectile dysfunction, as well as smoking 40 cigarettes or more/day. An alcohol consumption of up to 3 drinks/day was inversely associated with erectile dysfunction. Men reporting a medical diagnosis of diabetes or hypertension were more likely to present erectile dysfunction, while those with a history of heart disease, depression, benign prostatic hyperplasia or peptic ulcer were not. Depressive symptoms, as measured by CES-D scale, correlated significantly with erectile dysfunction prevalence (Table 4).

**Table 4:** Age-adjusted prevalence odds ratios for moderate or complete versus none or minimal erectile dysfunction of selected characteristics in 342 men, Santos, Brazil, 1999-2000

Characteristic	OR (95% CI) †
Marital status	
Never married	1 (reference)
Married or living with partner	1.2 (0.2 to 6.0)
Divorced, widowed, separated	3.1 (0.5 to 18.2)
Education	
Primary school completed or less	1 (reference)
High school (incomplete or graduated)	0.5 (0.1 to 1.7)
College (incomplete or graduated)	0.9 (0.4 to 2.0)
Religious affiliation (any)	
None	1 (reference)
Any	0.5 (0.1 to 2.6)
Obesity (body mass index > 30.0 kg/m^2^)	3.0 (1.3 to 7.4)
Monthly income < 3 minimum wages	1.7 (0.7 to 4.1)
Cigarette smoking	
Never smoked	1 (reference)
Past smokers	1.4 (0.6 to 3.4)
Current smokers	1.6 (0.6 to 4.3)
Heavy smoking (≥ 40 cigarettes/day)	3.1 (1.2 to 8.2)
Alcohol consumption	
None	1 (reference)
≤ 3 drinks/day	0.4 (0.2 to 0.9)
> 3 drinks/day	0.8 (0.3 to 2.4)
CES-D scale‡ (for each unit increase)	1.2 (1.0 to 1.3)
Medical conditions	
Diabetes	12.3 (4.5 to 33.5)
Hypertension	3.2 (1.5 to 6.7)
Heart disease	0.7 (0.2 to 2.9)
Benign prostate hyperplasia	0.8 (0.3 to 2.5)
Peptic ulcer	1.0 (0.4 to 2.6)
Depression	2.1 (0.6 to 7.1)

† Odds ratio — OR — (95% confidence interval — CI)

‡ Center for Epidemiological Studies depression scale.

### Multivariate model

Table 5 presents the results found in the multivariate analysis final model. Age was strongly correlated to erectile dysfunction (OR = 1.17; 95%, CI = 1.11-1.24) for each yearly increment in age. Heavy smoking remained associated with erectile dysfunction, while obesity and moderate alcohol consumption did not. Similar to the results of the age-adjusted bivariate analysis, self-reports of diabetes or hypertension were significantly associated with erectile dysfunction, and heart disease, depression, benign prostatic hyperplasia and peptic ulcer were not.

**Table 5:** Multivariate logistic regression model, prevalence odds ratios for moderate or complete versus none or minimal erectile dysfunction, Santos, Brazil, 1999-2000

Characteristic	OR (95% CI) †
Age, one-year increments	1.17 (1.11 to 1.24)
Heavy smoking, ≥ 40 cigarettes/day	3.6 (1.2 to 11.0)
Alcohol consumption	
≤ 3 drinks/day	0.4 (0.2 to 1.0)
> 3 drinks/day	0.7 (0.2 to 2.4)
CES-D scale‡ (for each unit increase)	1.13 (0.97 to 1.31)
Medical conditions	
Diabetes	8.9 (2.9 to 26.9)
Hypertension	2.3 (1.0 to 5.4)
Heart disease	0.5 (0.1 to 2.4)
Benign prostate hyperplasia	0.8 (0.2 to 2.8)
Peptic Ulcer	1.0 (0.3 to 2.9)
Depression	1.9 (0.5 to 7.0)

† Odds ratio — OR — (95% confidence interval — CI) for the variables included in the final model.

‡ Center for Epidemiological Studies depression scale.

### Health-seeking attitudes and behaviors with respect to erectile dysfunction

Subjects were probed about their attitudes toward seeking medical care for erectile dysfunction. First, they were asked whether, if they had erectile dysfunction, they would feel comfortable consulting a doctor or other health professional, and 82% said they would. Then, we asked "would you tell your doctor or any health professional about erectile dysfunction even if he/she did not ask about it?" and 94% said they would. However, of the 157 men actually reporting some degree of erectile dysfunction, only 16 (10.2%) had been treated for this condition.

## DISCUSSION

Our data indicate a high prevalence of erectile dysfunction in a sample of men from southeastern Brazil. Applying these rates to the Brazilian population, we estimate that currently over 9 million men aged 40 years or older present some degree of erectile dysfunction, and 3.5 million have moderate to complete dysfunction. Thus, the data suggests that erectile dysfunction is a common condition and a public health concern in Brazil.

The prevalence of erectile dysfunction (45.9%) we found is virtually equal to the prevalence of 46.2% reported in another population-based study of 1,170 men conducted in nine major cities in Brazil.^11^ These rates are also comparable to those reported in two studies done in the United States: 52% in the Massachusetts Male Aging Study,^10^ a survey in a random sample of 1,290 men aged 40 to 70 living in cities and villages near Boston, Massachusetts; and 46.3% in a population-based survey of approximately 1,650 men aged 50 to 76 in a rural zone in New York State.^3^

Similar erectile dysfunction rates were also found in France, where 39% of men aged 18 to 70 reported erectile dysfunction,^21^ and in Thailand, where a study using a sample of 1,250 men aged 40 to 70 revealed an erectile dysfunction prevalence of 37.5%.^22^

In our survey, as in other previous studies to date, such as the National Health and Family Life Survey (NHFLS),^23^ and the Massachusetts Male Aging Study,^10^ increasing age is correlated with both increasing prevalence of erectile dysfunction overall and with increasing severity. This remained true after controlling for all other significant correlates of erectile dysfunction.

Obesity, defined as a body mass index greater than 30.0 kg/m^2^, was a predictor of erectile dysfunction in the bivariate age-adjusted analysis, but not after adjusting for other potential confounders using multivariate analysis. The probabilities of erectile dysfunction were not dependent on body mass index or waist-to-hip ratio (an index of fat localization) in the Massachusetts Male Aging Study,^10^ although the prospective data from this study found that overweight exerted a strong independent effect on erectile dysfunction.^24^ In a study of 325 consecutive patients with erectile dysfunction, Chung et al.^25^ showed that obesity in itself does not seem to be an underlying factor, but does impose a risk of vasculogenic erectile dysfunction through the development of chronic vascular disease.

We found erectile dysfunction to be dependent on smoking dosage (cigarettes per day). In the Massachusetts Male Aging Study, no general effect was noted from current cigarette smoking, but the association of erectile dysfunction with certain risk factors was greatly amplified among current cigarette smokers.^10^ Cigarette smoking was also an independent risk factor for erectile dysfunction in a cross-sectional study including 4,462 US Army Vietnam-era veterans aged 31-49 years.^26^ Derby et al.^27^ showed smoking to be associated with increased erectile dysfunction risk in the prospective data from the Massachusetts Male Aging Study. The effect of smoking on erectile dysfunction may be mediated by the systemic changes induced by smoking, which include hypercoagulability, enhanced platelet aggregation, an imbalance between thromboxane and prostacyclin concentrations, and direct toxic effects on the vascular endothelium.^28^

Alcohol consumption of three drinks per day or less was inversely correlated with erectile dysfunction in our population in the bivariate, but not in the multivariate model. Rimm et al.,^29^ in the Health Professionals Follow-up Study, also found that moderate drinkers (one to two drinks per day) had a lower prevalence of erectile dysfunction than either non-drinkers or heavy drinkers. In the prospective data from the Massachusetts Male Aging Study, a change in heavy drinking status was not associated with reduced risk of erectile dysfunction,^27^ suggesting that chronic heavy alcohol consumption may have an irreversible effect on erectile function because of neurological damage.^30^

Erectile dysfunction has been shown to be more prevalent in diabetic individuals, with estimates ranging from 35 to 75%.^31-33^ We found moderate/complete erectile dysfunction in 53.8% of the participants with diabetes. There is controversy as to which of the several characteristics of diabetes is the direct causal agent of erectile dysfunction. Vascular disease is frequently mentioned^34^ and, in addition, autonomic neuropathy, gonadal dysfunction and vascular endothelium or neurogenic impairment of penile smooth muscle relaxation have also been implicated.^33,35^

Cardiovascular disease has been implicated in erectile dysfunction.^24,36^ Our data showed an association between erectile dysfunction and history of hypertension, but failed to demonstrate a significant relationship with histories of heart disease. As we had to rely on self-reporting, it is possible that medical conditions that are frequently asymptomatic or underdiagnosed have been underreported by subjects not aware of their existence. This would likely result in non-differential misclassification and bias the results towards the null.

Presence of depression symptoms was associated with erectile dysfunction in our study population, irrespective of age and the presence of other risk factors for erectile dysfunction, but the same was not true for the self-reporting of a diagnosis of depression. Similarly, Araujo et al.,^37^ using the Massachusetts Male Aging Study follow-up data, found a strong association between erectile dysfunction and the presence of depression symptoms. Despite the correlation between erectile dysfunction and depression being well documented, the causal relationship between the two is sometimes imprecise and most probably bi-directional, i.e. erectile dysfunction may follow depression and also, depression may be a consequence of this sexual dysfunction.^38,39^

When asked whether, hypothetically, they would feel comfortable consulting a health professional about erectile dysfunction, a high percentage of subjects (82%) said they would, but this was not reflected in the actual behavior of men with erectile dysfunction, of whom only (10.2%) had actually been treated. Educating physicians and laymen about the potential for treating erectile dysfunction would, we believe, lead to better treatment of this distressing condition.

### Merits and limitations

The main strength of this study was the assessment of erectile dysfunction presence by the subject himself in terms of a single global question (based strictly on criteria enunciated by the National Institutes of Health Consensus Conference on Impotence). The determination of erectile dysfunction by self-reporting has enabled our results to be compared with most of the epidemiological studies performed on erectile dysfunction. In addition, the anonymous and private approach to data collection allowed us to explore potential correlates of erectile dysfunction with minimal informational and observational bias. The response rate achieved (47.6%) in this study is remarkably higher than the average 1-5% rates reported for similar mail surveys in Brazil (Aglaé Diament, personal communication). Although this might be considered a strength, the large number of men who refused to participate may have biased our results, particularly if those losses were differential. Nevertheless, our estimates were consistent with a previous large population-based survey done elsewhere in Brazil,^11^ thus suggesting that they are reliable.

The main limitation of this study is that, due to its cross-sectional design, data collection was carried out solely via a self-completed questionnaire. Thus, the assessment of medical conditions was limited to self-reporting. Commonly asymptomatic or oligosymptomatic diseases may have been substantially underreported, which is likely to have resulted in non-differential classification error and attenuation of associations measured. Another limitation of our study is that, because of our sampling strategy, men in our survey tended to have higher educational attainment than the average Brazilian male. Since our data and previous studies^23^^,^^37^ have suggested that education is inversely related to erectile dysfunction prevalence, this imbalance in the composition of our study sample would, if anything, have biased our results towards a lower estimate of erectile dysfunction prevalence.

## CONCLUSIONS

Erectile dysfunction is a common condition in Santos, Brazil, whose prevalence and severity increases with age. The correlates of erectile dysfunction identified in our population are consistent with some, but not all, other previous studies on the epidemiology of this sexual dysfunction. Future research should include the objective assessment of the presence of medical conditions potentially associated with erectile dysfunction, particularly those frequently asymptomatic or underdiagnosed (e.g. diabetes, hypertension, heart disease etc.). In addition, studies on erectile dysfunction incidence based on populations are warranted in order to prospectively test the hypothesis generated by the associations encountered dysfunction in this cross-sectional study. We hope that correlates of erectile dysfunction identified here may help health professionals in the individual assessment of erectile dysfunction patients or, among patients presenting with erectile dysfunction, such correlates may induce investigation of underlying potential comorbidities.

Our data show that only one-tenth of the men with some degree of erectile dysfunction are being treated, although a majority say they would seek such treatment if they had an erectile disorder. Physicians need to be aware of a possible reluctance of men to discuss erectile dysfunction and to initiate discussions accordingly.
